# Exploring Dendrimer Nanoparticles for Chronic Wound Healing

**DOI:** 10.3389/fmedt.2021.661421

**Published:** 2021-05-11

**Authors:** Samuel Tetteh-Quarshie, Eric R. Blough, Cynthia B. Jones

**Affiliations:** ^1^Department of Pharmaceutical Science and Research, School of Pharmacy, Marshall University, Huntington, WV, United States; ^2^Department of Pharmacology, Physiology, and Toxicology, Joan C. Edwards School of Medicine, Marshall University, Huntington, WV, United States

**Keywords:** dendrimers, nanoparticles, silver nanocomposite-dendrimer, wound healing, chronic wounds

## Abstract

The United States spends billions of dollars to treat chronic wounds each year. Wound healing is complex in nature which involves several intricate multiphase processes that can be delayed for a number of reasons leading to the development of chronic wounds. Wound healing therapies range from topical preparations to surgical repair with treatment options that vary based on other underlying factors like co-infection, age, or co-morbidities such as diabetes. Historically, micelles and liposomes are some of the nanoparticle drug delivery systems explored to treat chronic wounds; however, recent data suggests that dendrimers have shown potential to rival these systems in treating chronic wounds as well as other diseases. This mini review examines advances in dendrimer nanoparticle drug delivery systems to treat chronic wounds.

## Introduction

It is estimated that the United States spends between $6 to $15 billion to treat chronic wounds every year and the average for treating diabetic foot wounds is estimated as high as $39 billion (1). Several factors may play a role in the delayed wound healing process, such as age, concurrent infections, obesity, medications, and diabetes (2), which lead to chronic wounds that need varying degrees of care. Treatment strategies for chronic wounds include topical antibiotics (3), debridement (4), occlusive dressing (3), skin grafts (3), negative pressure therapy (5), growth factors (3), hyperbaric oxygen (3), and more recently the application of dendrimer nanoparticles (6). Dendrimers are small artificial macromolecules designed with a large arrangement of a variety of functional groups (7). Although, recent data has suggested that dendrimers may be promising polymeric nanocarriers for a number of therapeutic agents (8) their potential use as drug delivery systems for wound healing remains to be determined.

## Dendrimer Unique Features

**Figure 1:**
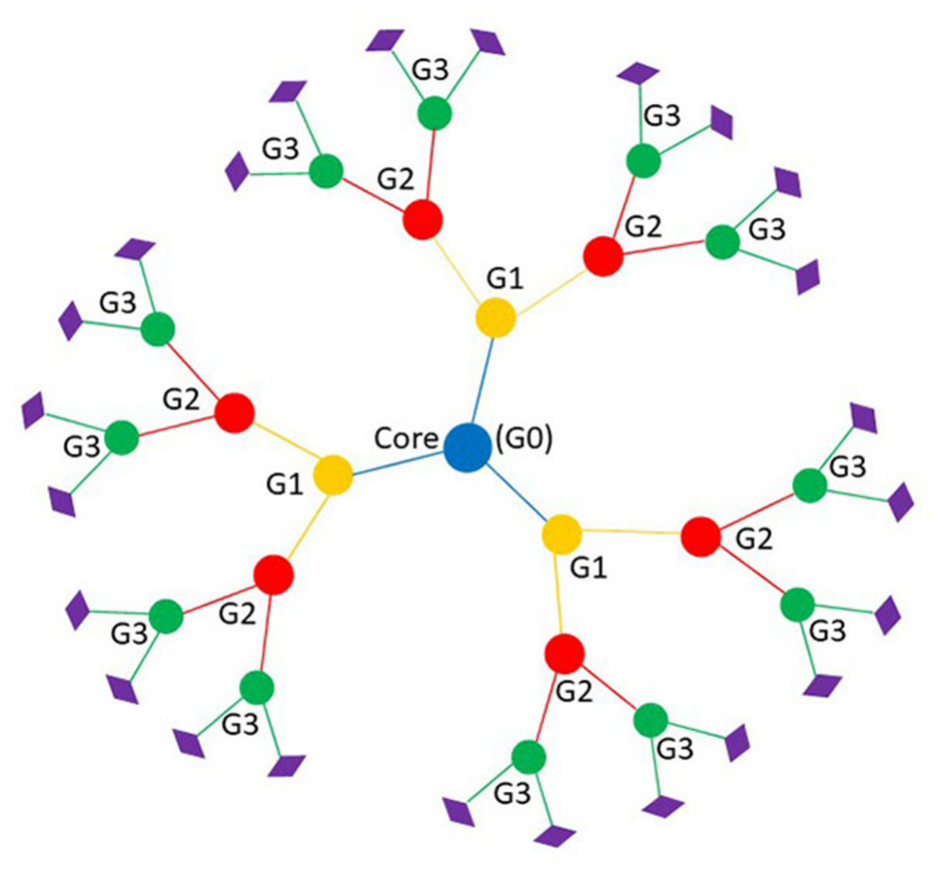
Basic dendrimer structure with three generations. Branches represent each generation; blue is the core of the dendrimer or generation zero, yellow is the first generation, red is the second generation, and green is third generation. Diamonds represent the peripheral functional group that make up the outer shell.

Dendrimers are a class of nano-sized (1–100 nm), three-dimensional globular molecules with distinct, uniform, and monodisperse structures resembling arborescent-like features (7, 9). The term dendrimer, coined by Tomalia, has its roots from the Greek words dendron, meaning “tree” or “branch” and meros meaning “part” (10, 11). The advancement of dendrimer biological utility is growing exponentially, with more than 100 families with 1000s of chemical modifications reported since its inception (11). Of all dendrimers studied to date, there are two at the forefront – Fréchet type polyether and Tomalia-type (Poly)amidoamine (PAMAM) – with encouraging promise (11). This is owning to their well-documented methods for synthesis as well as their commercial availability (12). Contrary to traditional polymers, dendrimers possess three distinct architectural components (Figure 1): it contains a centralized core, a middle or interior section made up of repeating branches which makes up each generation, and an outer shell defined by terminal functional groups (11).

The unique structure of dendrimers makes it an appropriate nanomaterial for drug delivery of molecules with solubility concerns or to target specific tissues, which is achieved by entrapping drugs within their void spaces, branches, or outer functional groups (13). Flexible open spaces within the voids allow entrapment of molecules in these spaces, and the functional groups forming the outer shell can be designed to interact with desired molecules of therapeutic choice (14). Drug entrapment is contingent upon the sizes and structures of the drug and dendrimer (13), the physicochemical properties of the interior units, and surface functional groups (14); therefore, understanding dendrimer structure is paramount for use as a drug delivery tool (13, 14). To that end, Maingi et al. (15) developed a validated dendrimer builder toolkit (DBT) that allows the design of customizable, theoretical dendrimer models prior to chemical synthesis. Additionally, the DBT can be used within the Assisted Model Building with Energy Refinement (AMBER) molecular dynamics software to simulate dendrimer interactions with biological molecules (15) and binding affinities and release profiles of guest molecules (16). It should be noted, PAMAM dendrimers exhibit strong binding to guest molecules at high pH values, which could be useful for controlled release at neutral pH values (16).

Two methods, divergent and convergent, are commonly used to chemically synthesize dendrimers. In the divergent approach, the dendrimer is synthesized from the core as the starting point and expanded generation by generation. Conversely, in the convergent approach the dendrimer is synthesized from the surface and ends up at the core (10). The attractive features of dendrimers are the acceptable excretory pathways, low probability to induce an immune response, and they have been demonstrated to exhibit low toxicity, which explain their widespread use in nanomedicine (17). In addition to these unique features, evidence shows that the surface of dendrimers provides an excellent platform for the attachment of specific cell targeting groups, solubility modifiers, and stealth moieties that reduce immunological interactions (17). In particular, the surfaces of dendrimers fitted with guanidine moieties have potential for use as cell penetrating peptides (18), which unlocks therapeutic opportunities to deliver numerous agents. Therefore, it is the capacity to bind these types of molecules onto the outer shell of dendrimers, in a specific and targeted approach, that makes them unique from other drug delivery system carriers (17).

## Dendrimers Use in Wound Healing

Recent advances in nanomedicine, especially in anticancer therapies, have driven formulation scientist to refocus their attention on how the newest class of nanoparticles, dendrimers, could be used in different clinical settings such as wound healing (19). The distinct property of dendrimers that has led to their emerging role in nanomedicine (Table 1) is their unique shape. The ongoing developments of polyester, polyacetal, and other biodegradable dendrimers as carriers and therapeutics are promising novel drug delivery strategies for a variety of treatments (26). According to Kalomiraki et al. (27), dendrimers can encapsulate small size drugs, metals, or imaging moieties that can fit within their branches, and can interact *via* hydrogen bonding, lipophilicity, and charge interactions due to their spherical shape. In addition to shape, the ability of dendrimers to easily penetrate cell walls due to their size and lipophilicity, may make them ideal drug delivery systems for many therapeutic treatments (27). These distinctive characteristics have boosted research interest in employing dendrimers nanoparticles for wound healing.

**Table 1:** Biomolecular-dendrimer constructs and therapeutic uses.

**Dendrimer type**	**Therapeutic**	**Treatment mechanism**	**Model**	**Ref**
Antimicrobial peptide dendrimers	Biological bandage	Prevents *Pseudomonas aeruginosa* infection, and improves wound healing from deep degree burns.	Human umbilical vein endothelial Cells (HUVEC); human skin progenitor cells; keratinocytes; and adult fibroblasts	(20)
Gelatin polyamidoamine (PAMAM) dendrimer	Silver nanofiber	Inhibits bacteria growth and infection during wound healing.	Bacteria colonies: (Staphylococcus *aureus*, and Pseudomonas *aeruginosa*)	(21)
Silver-dendrimer nanocomposite	Silver nanoparticles	Reduces expression of inflammatory cytokines, with improved wound healing time.	Macrophage cells; and C57BL/6 N Mice	(22)
Vancomycin-Ag PAMAM dendrimers	Vancomycin-Ag nanoparticles	Effective against resistant bacterial pathogens without inducing resistance in susceptible strains.	Vancomycin-susceptible Staphylococcus. *aureus;* and BALB/c female mice	(23)
Peptide-dendrimer	Fibronectin derivatives	Provides faster re-epithelialization and contraction of dermal wounds, leading to an accelerated diabetic wound healing.	Male Kunming mice; and rat plasma	(24)
Hyaluronic Acid-ASI-PAMAM Dendrimers	Antioxidant	Increases GSH levels and reduces reactive oxygen species in diabetic wounds.	BJ and HaCaT cells; and mice	(25)

Wound healing is a complex process that can be divided into a series of stages: homeostasis, inflammation, proliferation, and remodeling (28). There are a variety of cells, cell signaling proteins, and non-cellular components within cells that all interact during the wound healing process (22). In an attempt to develop effective wound healing therapies, recent studies have focused on combining dendrimers to different polymeric molecules to improve bioavailability, reduce cytotoxicity, and enhance therapeutic delivery to wounds. In a study conducted by Abdel-Sayed et al. (20) found that polycationic dendrimers are capable of having anti-angiogenic effects on burn wounds. Peptide dendrimers with three generations were prepared with amino acid residues L-lysine (G3KL) and L-arginine (G3R) dispersed throughout the branches to form two anti-microbial polycationic dendrimers (AMPD) (29). It was reported the AMPDs, G3KL, and G3R were safely used in combination with biological bandages, made of progenitor skin cells, to prevent Pseudomonas aeruginosa infection and improve wound healing in keratinocytes and endothelial cells (20). This remarkable study shows the clinical relevance of dendrimers in wound dressing application; however, further research is warranted to better understand the mechanism(s) underlying the modulation of anti-angiogenic/angiogenic effects.

In another study conducted by Dongargaonkar et al. (21) found that incorporating silver into gelatin-dendrimer nanofiber constructs were effective in inhibiting bacterial growth during the wound healing process. These preliminary finding are particularly important because bacterial infection is commonly associated with delayed wound healing, thus this unique gelatin-dendrimer nanofiber construct could easily be tailored to deliver other biomolecular compounds needed for wound healing (21). In other works, Liu et al. (30) using a silver dendrimer nanocomposite, showed that silver nanocomposite (AgNPs), and silver nanocomposite-dendrimer (AgDNC), both have anti-inflammatory effects following lipopolysaccharide-induced inflammation *in vitro* (30). In addition, these same researchers also showed that *in vivo* AgDNC results in faster healing time when compared to AgNPs. Because chronic inflammation often results in delayed or even non-healing consequences, it is conceivable that silver-dendrimer constructs could play a critical role in inhibiting local inflammatory responses which may help accelerate wound healing (30). More research to further bolster this assertion is needed.

Recently, dendrimers were used to treat resistant bacterial infections and have shown promising results when PAMAM is conjugated to vancomycin and silver. Jiang et al. (23) combined silver nanoparticle dendrimers with vancomycin to create a dual conjugated anti-microbial dendrimer that effectively treated vancomycin resistant S. *aureus in-vitro* and *in-vivo* and did not induce S. *aureus* resistance after repeated application *in-vitro* (23). Additionally, the novel PAMAM dual conjugate suspension was efficacious against infected wounds in BALB/c female mice after 9 days as compared to 14 days with the traditional silver nanoparticle dendrimer conjugate (23). This study is clinically useful for topical delivery of antibiotics to treat wound infections because it may limit the high doses of vancomycin a patient receives and eliminates unwanted side effects from systemic absorption seen in conventional dosage forms.

Diabetic wounds linger for extended periods of time because they heal at a slower pace than normal wounds, which adds to the list of complications of diabetes mellitus (31). Even though, managing diabetic wounds in clinical settings is difficult, the emergence of nanomedicine, especially dendrimers, provides a promising future for clinical treatment. For instance, a team of researchers investigated the utility of dendrimer peptides on diabetic wounds. Deng and co-workers synthesized dendrimer peptides on three different lysine cores, ranging from single lysine to five lysine residues, attached to the short peptide Ac-PHSRN-NH2, which originates from the cell binding site of fibronectin (24). They found that treating diabetic wounds with different fibronectin derivatives of peptide dendrimers provided faster re-epithelialization, increased contraction of dermal wounds, and accelerated wound healing in diabetic mice (24). The group surmised results from their study points to wound closure activity being closely linked to dendrimer branching. Clinically, this finding is very important because epithelialization is an essential component of wound healing, and the ability of peptide dendrimers to accelerate this process is paramount to solving any wound healing dilemma in the clinical setting.

Zhang et al. (25) synthesized PAMAM dendrimers conjugated to hyaluronic acid (HA) by a matrix metalloproteinases (MMP-2) polypeptide substrate to treat diabetic wounds employing an antioxidative approach. Their dendrimers were used to carry Astragaloside (ASI), a natural saponin constituent from the dried roots of the plant, Astragalus, that possess antioxidant activity and is utilized in traditional Chinese medicine (32). Their dendrimers were successful in targeting MMP-2, which drastically increased levels of glutathione thus reducing levels of reactive oxygen species (ROS). Moreover, dendrimers loaded with ASI improved expression of genes responsible for normal wound healing and improved keratinocyte proliferation and migration at greater levels as compared with ASI alone (25). These findings ultimately promote wound healing and demonstrate the superior therapeutic advantage of dendrimers as nanocarriers.

## Discussion

The rare properties of dendrimers make them ideal nanoparticle drug delivery systems for many treatment modalities. Dendrimers have the capability to interact with other particles by hydrogen bonding, lipophilicity, or charged interactions, which provides flexibility for use with many therapeutics. These properties are valuable in their utility to deliver drugs that exhibit poor solubility, decrease drug toxicity, or improve stability for drugs that are susceptible to various degradative processes (33). Treatment for wound healing is a difficult and expensive process which requires treatment strategies to ease the burden and reduce costs. Dendrimer nanoparticles have shown promise to treat wound infections (21), reduce inflammation in burn wounds (30) and relieve wound oxidative stress (25). As the incidence of diabetes steadily increases, so does the number of chronic wounds (34), which supports the need for novel treatment. Although, more research is required, the endless possibilities of utilizing dendrimer nanoparticles as a new tool for the treatment of chronic wounds is an exciting prospect that could advance therapeutic wound healing.

## Author Contributions

ST-Q, EB, and CJ: drafting the paper or revising it critically. All authors have read and approved the final submitted manuscript.

## Conflict of Interest

The authors declare that the research was conducted in the absence of any commercial or financial relationships that could be construed as a potential conflict of interest.
